# Effect of Non-Vital Bleaching on the Durability of Resin–Dentin Bond with an Ethanol-Based Etch-And-Rinse Adhesive

**DOI:** 10.3390/biomimetics3040035

**Published:** 2018-11-06

**Authors:** Alireza Boruziniat, Atefeh Atoufi, Zafer Chehreli, Majid Akbari, Mahshid Gifani

**Affiliations:** 1 Dental Research Center, Mashhad University of Medical Sciences, P.O. Box 9177948959, Mashhad, Iran; borouziniata@mums.ac.ir (A.B.); Akbarim@mums.ac.ir (M.A.); 2 Department of Pediatric Dentistry, Faculty of Dentistry, Sihhiye 06100, Turkey; zcehreli@hacettepe.edu.tr; 3 Department of Pediatric Dentistry, School of Dentistry, Mashhad University of Medical Sciences, P.O. Box 9177948959, Mashhad, Iran; Gifanimh951@mums.ac.ir

**Keywords:** teeth bleaching, matrix metalloproteinases, sodium ascorbate, dentin bonding agent

## Abstract

To evaluate the bleaching procedure and application of sodium ascorbate on dentin bond durability, the enamel surface of intact human third molars (*n* = 18) were removed, and the teeth were randomly divided into two groups for immediate and six-month bond strength evaluation. The specimens were further assigned into three subgroups according to treatment procedure, as follows: subgroup A, no bleaching (positive control) was performed prior to the etch-and-rinse bonding with single bond and subsequent build-up with Z250 composite; subgroup B, the samples were bleached with 20% carbamide peroxide 6 h/day for five consecutive days prior to bonding; and subgroup C, bleaching was performed as in subgroup B, after which 10% sodium ascorbate was applied on dentin surface for 10 min before the bonding procedures. A microtensile bond strength test was performed and the failure modes were evaluated under a stereomicroscope. The data were analyzed using two-way analysis of variance (ANOVA) and Tukey’s post-hoc tests with a level of significance of 0.05. Bleaching significantly decreased the immediate and six-month bond strength. The application of sodium ascorbate had no significant effect on the immediate and six-month bond strength of bleached specimens. We conclude that the bleaching procedure may decrease the durability of the resin–dentin bond of the tested etch-and-rinse adhesive.

## 1. Introduction

Bleaching is an established method to manage tooth discoloration via the application of oxidizing agents. Despite several advantages, different studies have demonstrated several drawbacks of this technique, such as pulp inflammation [1], changes in tooth structure [2], tooth sensitivity [1], external root resorption [3], and a decrease in enamel and dentin bond strength [4,5]. Interference with resin infiltration and polymerization, as well as an alteration of the tooth structure are the main reasons for up to a 67% reduction in bond strength [4,6,7]. Cavalli et al. [8] and Josey et al. [9] reported that the adhesive interface of bleached specimens demonstrated shallow resin infiltration, scattered resin tags with ill-defined borders, and the existence of granular areas of air bubbles. Different methods have been suggested to improve bond strength, which vary from postponing adhesive restorative procedures for up to three weeks, to the use of antioxidant agents or organic solutions such alcohol or acetone [10,11,12,13,14,15,16,17,18,19].

Recently, Toledano et al. [20] and Sato et al. [21] demonstrated that bleaching agents may activate matrix metalloproteinases (MMPs), which, in turn, can destroy exposed collagen fibrils and degrade the resin–dentin bond. Matrix metalloproteinase are calcium and zinc dependent endopeptidasesthat exist in dentin and have a role in normal and pathologic tissue remodeling [22]. Under normal conditions, they exist in pro-enzyme or inactive form, and can be activated by heat, chemical agents, reactive oxygen, free radicals, and acidic pH caused by phosphoric acid etching [21,23,24,25,26]. In etch-and-rinse adhesives, inadequate resin penetration into etched dentin can leave exposed collagen fibrils at the base of the hybrid layer [27,28], which can be degraded by MMPs and can lead to a decrease in the durability of the bond to dentin [25,29,30].

While several studies have demonstrated that bleaching agents decrease the immediate bond strength to tooth structure [4,6,7], no evidence regarding the long-term bonding effectiveness to bleached dentin has been discovered. Considering these observations, the aim of this study was to evaluate the effect of the bleaching procedure on dentin bond durability. The null hypotheses of this study were: (i) the bleaching procedure would not affect the durability of resin–dentin bond; and (ii) the application of sodium ascorbate would not improve bond strength.

## 2. Materials and Methods

### 2.1. Sample Selection and Preparation

This study was approved by the Ethics Committee of Mashhad University of Medical Sciences, Mashhad, Iran (code no.: IR.MUMS.REC.1392.88). Eighteen freshly-extracted intact human third molars were collected under informed consent. Following the debridement of the soft tissue remnants, the teeth were disinfected with 1% chloramine-T (Merck, Darmstadt, Germany) and were stored in saline at room temperature, until the actual experiments (two weeks). The occlusal enamel was sectioned off using a slow-speed water cooled diamond saw (Drendel+Zweiling Diamant GmbH, Berlin, Germany), and the teeth were mounted in self-cure acrylic blocks 1 mm below the cementoenamel junction, with their long axis perpendicular to the horizontal plane. Then, the dentin surfaces were gently ground flat using 400- and 600-grit silicon carbide papers (Shanghai Hangli Industrial Co., Ltd., Shanghai, China) under running water. The samples were randomly divided into two groups for immediate and six-month bond strength evaluation. In each group, the specimens were further assigned into three subgroups, with respect to the following bleaching procedures: (A) no bleaching (positive control); (B) bleaching with 20% carbamide peroxide; and (C) bleaching with 20% carbamide peroxide, followed by surface treatment with 10% sodium ascorbate for 10 min, before the bonding procedures.

### 2.2. Bonding Procedure

In each specimen, the dentin was etched for 15 s using 35% phosphoric acid gel (ScotchbondEtchant, 3M ESPE, Saint Paul, MN, USA), rinsed with distilled water for 10 s, and air dried. An ethanol-based etch-and-rinse adhesive (Single Bond Adhesive 3M ESPE) was applied on the dentin surfaces according to the manufacturer’s instructions, and was polymerized using a light-emitting diode (LED)-curing unit (Blue Phase C8, Ivoclar Vivadent, Schaan, Lichtenstein) at 800 mW/cm^2^ for 20 s. The intensity of the irradiation was confirmed with a radiometer before application. The resin composite build-up was accomplished using two 2 mm increments Z250 (Shade A1, 3M ESPE), with each increment being light-cured for 40 s. The specimens were post-cured from each surface for 40 s.

### 2.3. Bleaching Procedure

In subgroups B and C, the specimens were subjected to bleaching with 20% carbamide peroxide gel (Opalescence, Ultradent, South Jordan, UT, USA). Each specimen received 0.1 mL of bleaching gel for 6 h/day, for five days. After each 6 h application, the gel was washed off, and the samples were stored in fresh distilled water at 37 °C until the next bleaching cycle. After the bleaching procedure, the samples were washed off carefully and stored in fresh distilled water at 37 °C.

### 2.4. Pretreatment with Ascorbate Solution

In subgroup C, a 10% sodium ascorbate solution (Daejung Chemicals & Metals Co., Ltd., Siheung, Korea) was applied on the dentin surfaces for 10 min immediately after the bleaching procedure. The solution was renewed every 20 s. After 10 min, the sodium ascorbate solution was rinsed away using distilled water for 30 s.

### 2.5. In Vitro Ageing

To evaluate the short-term durability of resin–dentin bonds, randomly selected specimens were stored in saline for six months at room temperature [1,24,31]. The saline was refreshed weekly.

### 2.6. Microtensile Bond Strength and Failure Modes Evaluation

The specimens were serially sectioned perpendicular to the adhesive interface in order to obtain slices with 10 mm length and a cross-sectional area of approximately 1 mm. Five intact beams were obtained from each sample. The microtensile bond test was performed at a cross-head speed of 1 mm/min. The failure modes were determined at 40 × magnification using a stereomicroscope (Zeiss, Oberkochen, Germany), and were recorded as adhesive, cohesive in dentin or composite, and mixed.

### 2.7. Statistical Analysis

The data were processed using SPSS version 11.5 software (SPSS Inc., Chicago, IL, USA). The normal distribution of the data was confirmed using the Kolmogorov–Smirnov test, and the data were analyzed using two-way analysis of variance (ANOVA) and Tukey’s post-hoc tests with the level of significance set at 0.05.

## 3. Results

**Figure 1:**
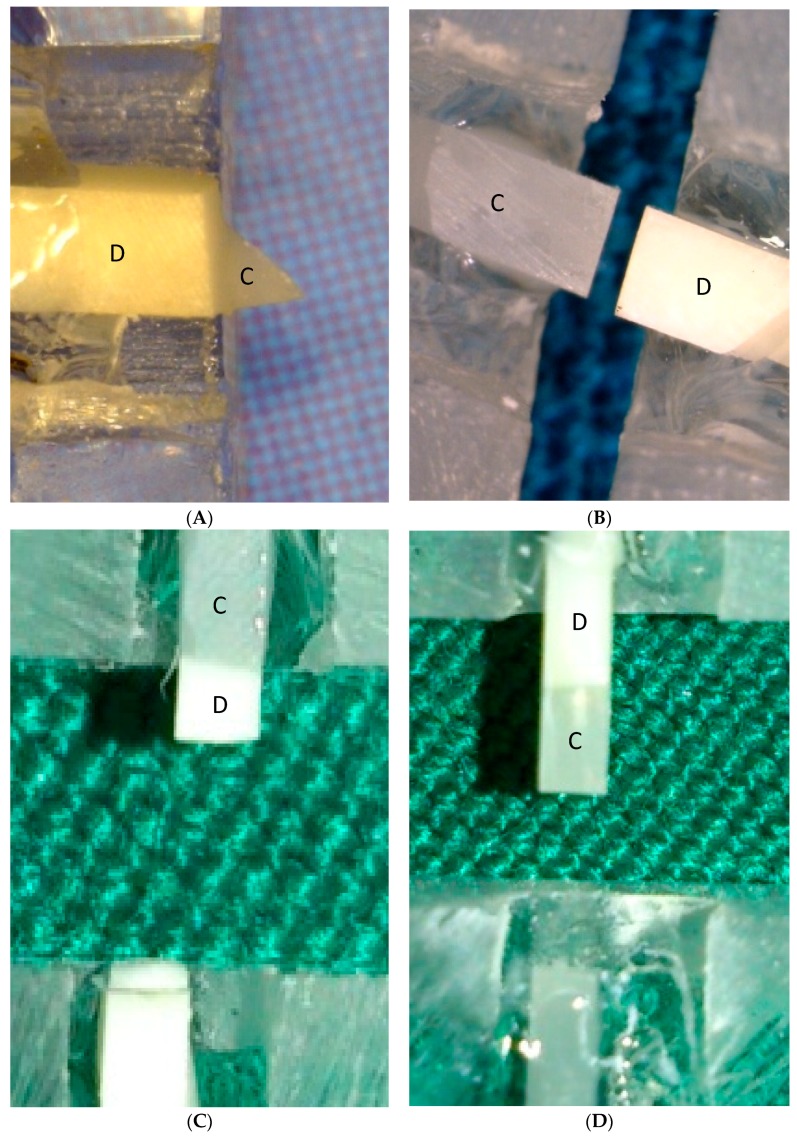
Modes of failure of the experimental groups. (**A**) Mixed failure; (**B**) adhesive failure; (**C**) cohesive failure of dentin; (**D**) cohesive failure of composite. C: Composite; D: Dentin.

The mean microtensile bond strength values are presented in Table 1. The two-way ANOVA indicated that both variables (treatment groups and time) had a statistically significant effect on the bond strength (*p* < 0.001). The Tukey’s post-hoc test indicated that the bond strength of the subgroup A was significantly higher than the two experimental subgroups (B and C) (*p*-value < 0.001), and there was no statistically significant difference between the bond strength of the two experimental groups (*p*-value = 0.279). The results of the failure modes evaluation are presented in Table 2. Adhesive failure was the most frequently observed failure mode, followed by mixed failure (Figure 1).

## 4. Discussion

The presented findings allow us to reject the first null hypothesis, because bleaching with 10% carbamide peroxide significantly reduced the durability of the resin–dentin bond. This finding may be attributed to the activation of MMPs by the release of free radicals and is consistent with previous work by Toledano et al. [20] and Sato et al. [21], who showed that bleaching agents can activate MMPs. Here, carbamide peroxide was used for bleaching, as it has been shown to promote the highest levels of collagenolytic activity, which may remain for up to four weeks [20]. The bleaching application period was chosen according to a previous study [14]. Although the present results and previous studies demonstrate that the bleaching procedure can reduce the immediate bond strength to tooth structure via the inhibition of resin infiltration and polymerization, or altering the tooth structure [4,5,6,7], it seems that this procedure can also reduce the bond durability as a result of extended MMP activity [20]. The interference with resin infiltration and the polymerization of resin may remain more exposed to collagen fibrils at the base of hybrid layer in comparison with the control group. As more exposed collagen fibrils exist, more degradation may occur via hydrolysis or MMPs activation. In the current study, about a 25% reduction of bond strength was observed in the control group after six months of storage, but in the bleached group this was about 45%. In addition to the effects of carbamide peroxide, hydrolytic degradation and MMP activation by the acid etching process may also affect the durability of the bond [24,30,32], both of which may have contributed to the significant reduction of the bond strength after six months ageing in vitro, especially in the control group. Toledano et al. [20] and Sato et al. [21] demonstrated that bleaching may increase MMP activity by evaluation of collagen degradation product liberation or by assessment of proteolytic activities. In the current study, MMP activity was not evaluated although our findings suggest the possible role of MMPs in the durability of the resin–dentin bond.

In the present study, specimens were subjected to water storage for the evaluation of short-term bond durability, as this method appears to be the most valid approach for the ageing of restorations in vitro [1,24,31]. Adhesive failure was the most frequent failure mode among the experimental groups. These results are in line with those of El-din et al. [33] and Gurgan et al. [34], who concluded that there is no relationship between bond strength and failure mode. In contrast, Moule et al. [35] concluded that the application of bleaching materials slightly increases the cohesive failure rate.

Several studies have reported that the application of sodium ascorbate for 10 min can improve the immediate bond strength to the bleached tooth structure [18,36,37,38,39]. These findings are in stark contrast with the present results and, thus, the second null hypothesis was accepted. Lai et al. [5] reported that antioxidant agents such as sodium ascorbate should be applied for at least one-third of the entire duration of the bleaching process, in order to neutralize free radicals and to improve the bond strength. For 10% sodium ascorbate, they suggested an application time of 3 h [5]. Dabas et al. [37] suggested that sodium ascorbate should be applied for at least 60 min. Apparently, both durations are not practical in a clinical setting. By contrast, Turkun et al. [36] reported that the application of sodium ascorbate for 10 min is sufficient to improve the immediate bond strength, provided that the solution is refreshed during the application. Although the sodium ascorbate was refreshed every 20 s in the present study, the bond strength did not significantly improve in comparison with the control group. It should be noted that the samples were rinsed with distilled water after the application of sodium ascorbate, so as to avoid the precipitation of salt on the tooth structure. Presumably, this procedure prevented the extended contact time of sodium ascorbate on the tooth surface, which might have helped neutralize the free radicals and inhibit activation of MMPs. On the other hand, the extended contact time replicates the scenario of Lai et al. [5] and Dabas et al. [37], and may be impractical in many clinical settings.

Because of the limitations of this study, only one bleaching agent was used, so these results may not be applicable to all bleaching materials. Also, it should be considered that other factors such as the type of adhesive systems, or the composite and light-curing method may affect the bond durability in clinical situations.

## 5. Conclusions

With the limitations of this in vitro study, it can be concluded that the bleaching of dentin with 20% carbamide peroxide significantly reduces the six-month bond strength or bond durability in comparison with the control group by activation of MMPs. Furthermore, the application of 10% sodium ascorbate cannot restore the compromised bond strength. Further research is necessary in order to evaluate alternative methods/chemicals to reverse the bond strength of adhesives to bleached dentin.

## Figures and Tables

**Table 1:** The microtensile bond strength values of the test groups.

Groups	Debonding Time	*n*	Mean (MPa)	Standard Deviation	Standard Error
A (Control)	24 h	15	34.60 ^a^	6.95	1.79
	6 months	15	26.16 ^b^	12.03	3.10
B (Bleaching)	24 h	15	19.34 ^bc^	5.87	1.51
	6 months	15	10.78 ^d^	3.09	0.79
C (Bleaching + SA)	24 h	15	26.04 ^b^	6.39	1.65
	6 months	15	17.74 ^dc^	4.22	1.08

SA: Sodium ascorbate; 24 h: Immediate bond strength; 6months: Bond strength after six months storage. Values with different superscript letters denote a statistically significant difference at *p* < 0.05.

**Table 2:** Distribution of failure mode in experimental groups.

Mode of Failure	Debonding Time	A (Control)	B (Bleaching)	C (Bleaching + SA)
Adhesive	24 h	9	13	13
	6 months	10	13	11
Mixed	24 h	3	2	2
	6 months	4	2	4
Cohesive within composite	24 h	2	0	0
	6 months	1	0	0
Cohesive within dentin	24 h	1	0	0
	6 months	0	0	0

SA: Sodium ascorbate; 24 h: Immediate bond strength; 6 months: Bond strength after six months storage.
